# Constitutive expression and cell-surface display of a bacterial β-mannanase in *Lactobacillus plantarum*

**DOI:** 10.1186/s12934-019-1124-y

**Published:** 2019-04-25

**Authors:** Hoang-Minh Nguyen, Mai-Lan Pham, Elena Maria Stelzer, Esther Plattner, Reingard Grabherr, Geir Mathiesen, Clemens K. Peterbauer, Dietmar Haltrich, Thu-Ha Nguyen

**Affiliations:** 1 Department of Biotechnology, The University of Danang-University of Science and Technology, 54 Nguyen Luong Bang, Danang, Vietnam; 2 Food Biotechnology Laboratory, Department of Food Science and Technology, BOKU-University of Natural Resources and Life Sciences Vienna, Muthgasse 18, 1190 Vienna, Austria; 3 Department of Biotechnology, BOKU-University of Natural Resources and Life Sciences Vienna, Muthgasse 18, 1190 Vienna, Austria; 4 Faculty of Chemistry, Biotechnology and Food Science, Norwegian University of Life Sciences (NMBU), N-1432 Ås, Norway

**Keywords:** Cell-surface display, Whole-cell biocatalyst, *Lactobacillus plantarum*, Mannanase, Lipoprotein anchor, Constitutive promoter, SlpA, Pgm

## Abstract

**Background:**

Lactic acid bacteria (LAB) are important microorganisms in the food and beverage industry. Due to their food-grade status and probiotic characteristics, several LAB are considered as safe and effective cell-factories for food-application purposes. In this present study, we aimed at constitutive expression of a mannanase from *Bacillus licheniformis* DSM13, which was subsequently displayed on the cell surface of *Lactobacillus plantarum* WCFS1, for use as whole-cell biocatalyst in oligosaccharide production.

**Results:**

Two strong constitutive promoters, Pgm and SlpA, from *L. acidophilus* NCFM and *L. acidophilus* ATCC4356, respectively, were used to replace the inducible promoter in the lactobacillal pSIP expression system for the construction of constitutive pSIP vectors. The mannanase-encoding gene (*manB*) was fused to the N-terminal lipoprotein anchor (Lp_1261) from *L. plantarum* and the resulting fusion protein was cloned into constitutive pSIP vectors and expressed in *L. plantarum* WCFS1. The localization of the protein on the bacterial cell surface was confirmed by flow cytometry and immunofluorescence microscopy. The mannanase activity and the reusability of the constructed *L. plantarum* displaying cells were evaluated. The highest mannanase activities on the surface of *L. plantarum* cells obtained under the control of the Pgm and SlpA promoters were 1200 and 3500 U/g dry cell weight, respectively, which were 2.6- and 7.8-fold higher compared to the activity obtained from inducible pSIP anchoring vectors. Surface-displayed mannanase was shown to be able to degrade galactomannan into manno-oligosaccharides (MOS).

**Conclusion:**

This work demonstrated successful displaying of ManB on the cell surface of *L. plantarum* WCFS1 using constitutive promoter-based anchoring vectors for use in the production of manno-oligosaccharides, which are potentially prebiotic compounds with health-promoting effects. Our approach, where the enzyme of interest is displayed on the cell surface of a food-grade organism with the use of strong constitutive promoters, which continuously drive synthesis of the recombinant protein without the need to add an inducer or change the growth conditions of the host strain, should result in the availability of safe, stable food-grade biocatalysts.

## Background

β-Mannanase (EC 3.2.1.78) catalyzes random hydrolysis of β-1,4-mannosidic linkages in the main chain of β-1,4-mannans, glucomannans, and galactomannans into manno-oligosaccharides (MOS) [1]. Notably, MOS have been shown to have health-promoting effects on both humans and livestock [2–4]. Therefore, many studies are focusing on the production of MOS in an economic, efficient and substantial way [3, 5–8]. Currently, there is an increasing amount of attention on the utilization of β-mannanase for bioconversion of abundant, inexpensive agricultural by-products such as copra meal, or coffee extract into bioactive MOS due to its strong activity of substrate degradation [9–11]. It has been shown that β-mannanase was efficiently produced and secreted in *L. plantarum*, a well-studied probiotic member of the lactobacilli, using the inducible promoter-based pSIP expression vectors [5]. However, the methods based on free enzymes would involve extensive downstream activities such as disruption, purification, and loss of enzyme after reaction [3, 12, 13]. Moreover, chemical immobilization of the protein also requires a laborious and detrimental procedure and there are some drawbacks such as low recovery rate of enzyme activity, the gradual loss of enzyme during the reaction process or large mass transfer resistance between some immobilized enzymes and substrates [14]. In the so-called genetic immobilization, proteins of interest, which are fused with the anchoring motifs, are synthesized and subsequently anchored on the bacterial cell surface, hence the immobilized enzymes can be easily obtained from the cultivations, using the bacterial biomass as the immobilization matrix [15–18]. This method actually overcomes many limitations of free enzymes or conventional immobilization by letting the cells do the whole procedure [19]. In addition, surface-displayed enzymes possess high tolerance or stability in harsh conditions, especially when proteins are embedded in the bacterial cell wall, and can be reused in several process cycles [18, 19].

Thorough understanding about the mechanisms of different anchoring motifs would be greatly useful for the development of cell surface systems in *Lactobacillus* to be used for the immobilization of the enzymes of interest for food and biotechnological applications. In principle, a heterologous protein can be attached onto lactobacillal cell envelope via two major strategies: via covalent attachment to the cell membrane or the cell wall, which can be attained by lipoprotein anchors or by employing the sortase pathway, or non-covalently using a protein domain such as LysM-derived motifs that interacts strongly with components of the cell wall or the membrane [12, 20, 21]. We have recently reported the expression and display of a mannanase from *Bacillus licheniformis* and a chitosanase from *Bacillus subtilis* on the cell surface of *L. plantarum* WCFS1 using an inducible lactobacillal expression system and two anchoring motifs of *L. plantarum* for covalent attachment to the cell surface, either via an N-terminal lipoprotein anchor or a C-terminal cell wall anchor [22].

When aiming at food-related applications, the use of inducible lactobacillal expression vectors, which require the addition of synthetic inducer (IP) into the cultivation medium, might not be a preferred choice [23]. In addition, the inducible expression system does not fit with the applications related to in situ production of delivery of therapeutic or enzymatic compounds in the human body [24]. Therefore, the use of constitutive expression vectors for the production of proteins at a desirable level would be an alternative. In this study, we investigated two constitutive promoters, Pgm, the promoter of a phosphoglycerate mutase (*pgm*) from *L. acidophilus* NCFM, and SlpA, the promoter of a well-characterized S-layer protein SlpA of *L. acidophilus* ATCC 4356, for the expression, secretion and display of a β-mannanase from *Bacillus licheniformis* DSM13 in *L. plantarum* WCFS1 using a lipoprotein anchor Lp_1261 from *L. plantarum* [25, 26]. These constitutive promoters were demonstrated as strong promoters for the intracellular production of heterologous proteins in lactobacilli [25, 26], but the use of these promoters for extracellular expression and display of heterologous proteins on the bacterial cell surface has not been reported. Thus, the evaluation of the functionality of these constitutive promoters for surface display of an active mannanase might pave the way to the development of safe, food-grade whole cell biocatalysts that are relevant for the production of health-promoting oligosaccharides.

## Results

### Constitutive expression of ManB in *L. plantarum*

**Fig. 1:**
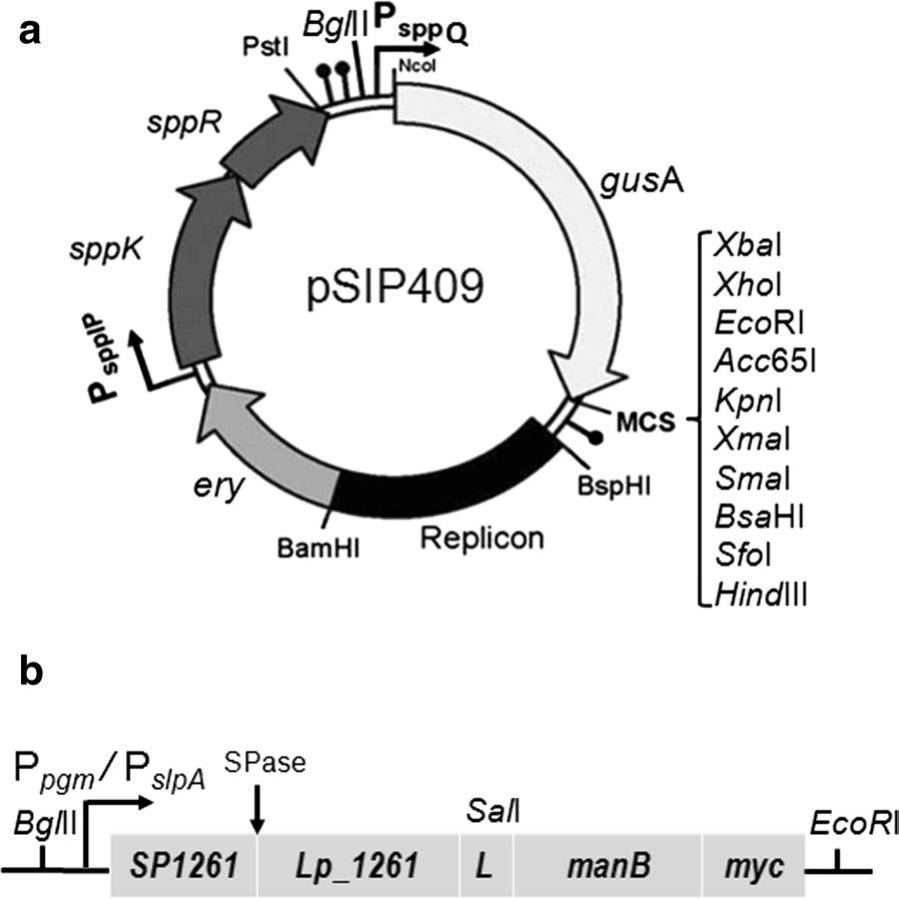
The construction of the constitutive expression vectors for N-terminal lipoprotein anchoring of mannanase (ManB) in *L. plantarum*. **a** Schematic overview of the pSIP409 expression vector, the starting point for construction. The *sppK* and *sppR* genes encode the proteins in the two-component regulatory system. The gene of interest is placed under control of a strong inducible bacteriocin promoter P_*sppQ*_-sakacin Q. The multiple cloning site (MCS), the replicon region consists of two determinants pUC(pGEM)-ori for *E. coli* and 256_rep_ for *L. sakei* and *L. plantarum*. The selection marker is the erythromycin resistance gene (*ery)*, the lollipops indicate transcription terminators, L indicates the *Sal*I-linker sequence. **b** Schematic overview of the constitutive expression cassette for N-terminal lipoprotein anchoring of mannanase (ManB) with *Bgl*II and *Eco*RI cloning sites. The inserts containing the *manB* sequence were fused with a myc tag for protein detection. P_*pgm*_ and P_*slpA*_: the promoters of a phosphoglycerate mutase (*pgm*) from *L. acidophilus* NCFM and a S-layer protein SlpA of *L. acidophilus* ATCC 4356, respectively; SP1261 and Lp_1261: a signal peptide and a lipoprotein anchor; SPase: lipobox withSignal Peptidase II cleavage site (SPase); L: linker (*Sal*I site)

**Fig. 2:**
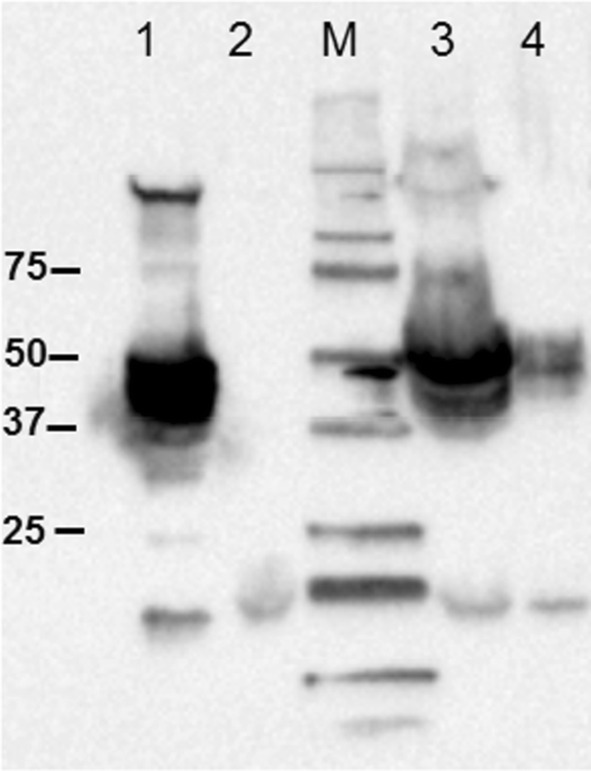
Western blot analysis of cell-free extracts from *L. plantarum* cells harboring constitutive expression vectors for lipoprotein anchoring of mannanase (ManB) (1) pSIP_1261ManB: *L. plantarum* harboring inducible expression vector for lipoprotein anchoring of ManB as positive control (protein size 51 kDa); (2) pEV: *L. plantarum* harboring an empty vector as negative control; (3) pSlpA_1261ManB (expected protein size 51 kDa); (4) pPgm_1261ManB (expected protein size 51 kDa). Lane M indicates molecular mass markers

Inducible lactobacillal expression vector pSIP409 was selected as a starting point for the construction of the vectors for constitutive expression, secretion and anchoring of mannanase (ManB) to cell membrane of *L. plantarum*. The strong inducible bacteriocin promoter P_*sppQ*_-sakacin Q in pSIP409 was replaced by two constitutive promoters, Pgm and SlpA (Fig. 1). In order to analyse the production of the target enzyme, cells were harvested 4 h after inoculation and subsequently disrupted by glass beads. Western blot analysis of the crude, cell-free extracts was performed using anti-Myc antibodies for detection of the mannanse. Figure 2 shows the presence of mannanase at expected molecular weight of ~ 51 kDa. Moreover, the crude protein extract from the strain harboring pSlpA_1261ManB showed a more prominent band than the one obtained from the pPgm_1261ManB carrying strain, indicating higher production of mannanase using the SlpA promoter compared to the Pgm promoter. The enzymatic activities of ManB-displaying cells obtained with the strains carrying the plasmids pSlpA_1261ManB and pPgm_1261ManB (see section below) also support this observation.

### Cell-surface display of ManB in *L. plantarum*

**Fig. 3:**
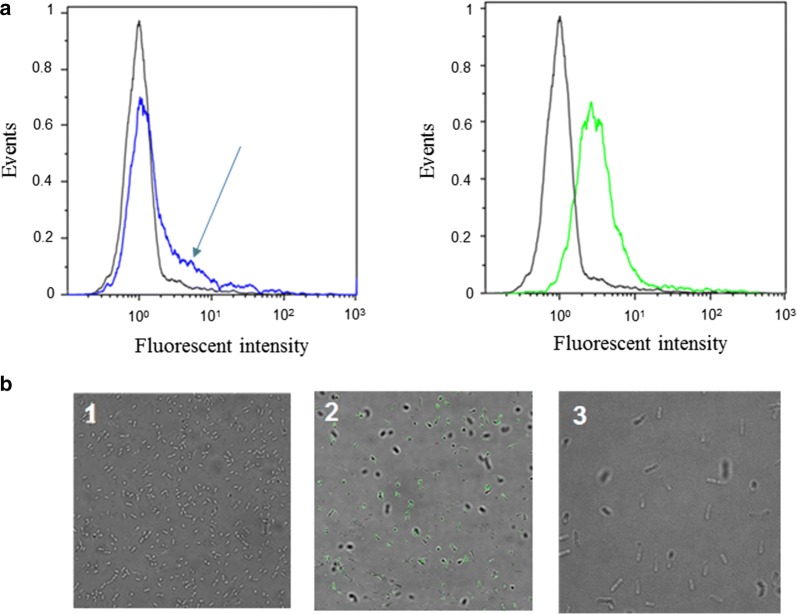
Surface localization of ManB in *L. plantarum* cells analyzed by flow cytometry (**a**) and immunofluorescent microscopy (**b**). *L. plantarum* strains harboring ManB-encoding plasmids are depicted by different colors in the flow cytometry histograms (**a**) and different numbers in the micrographs (**b**): pPgm_1261Man (blue, 1, an arrow indicates the ‘shoulder’ of surface mannanase detected by slight shift in the fluorescent signal); pSlpA_1261Man, (green, 2); *L. plantarum* harboring an empty vector pEV was used as negative control (black, 3)

To display ManB on the cell surface of *L. plantarum*, the enzyme was N-terminally anchored to the cell membrane employing a 75-residue lipoprotein anchoring sequence derived from the Lp_1261 protein of *L. plantarum* [27]. Flow cytometry analysis confirmed surface display of the mannanase in both recombinant bacteria as indicated by the shifts in the fluorescence signals observed for both strains compared to the negative control strain, even though only slight shift in the fluorescent signal was detected for the strain harboring pPgm_1261ManB (Fig. 3a). Immunofluorescence microscopy confirmed surface exposure of the mannanase in strain carrying pSlpA_1261ManB, while no signal was obtained with pPgm_1261ManB (Fig. 3b). Western blotting using the strain carrying pPgm_1261ManB showed ManB production, however, the observation of surface exposure was ambiguous.

### Enzymatic activity of ManB-displaying cells

**Fig. 4:**
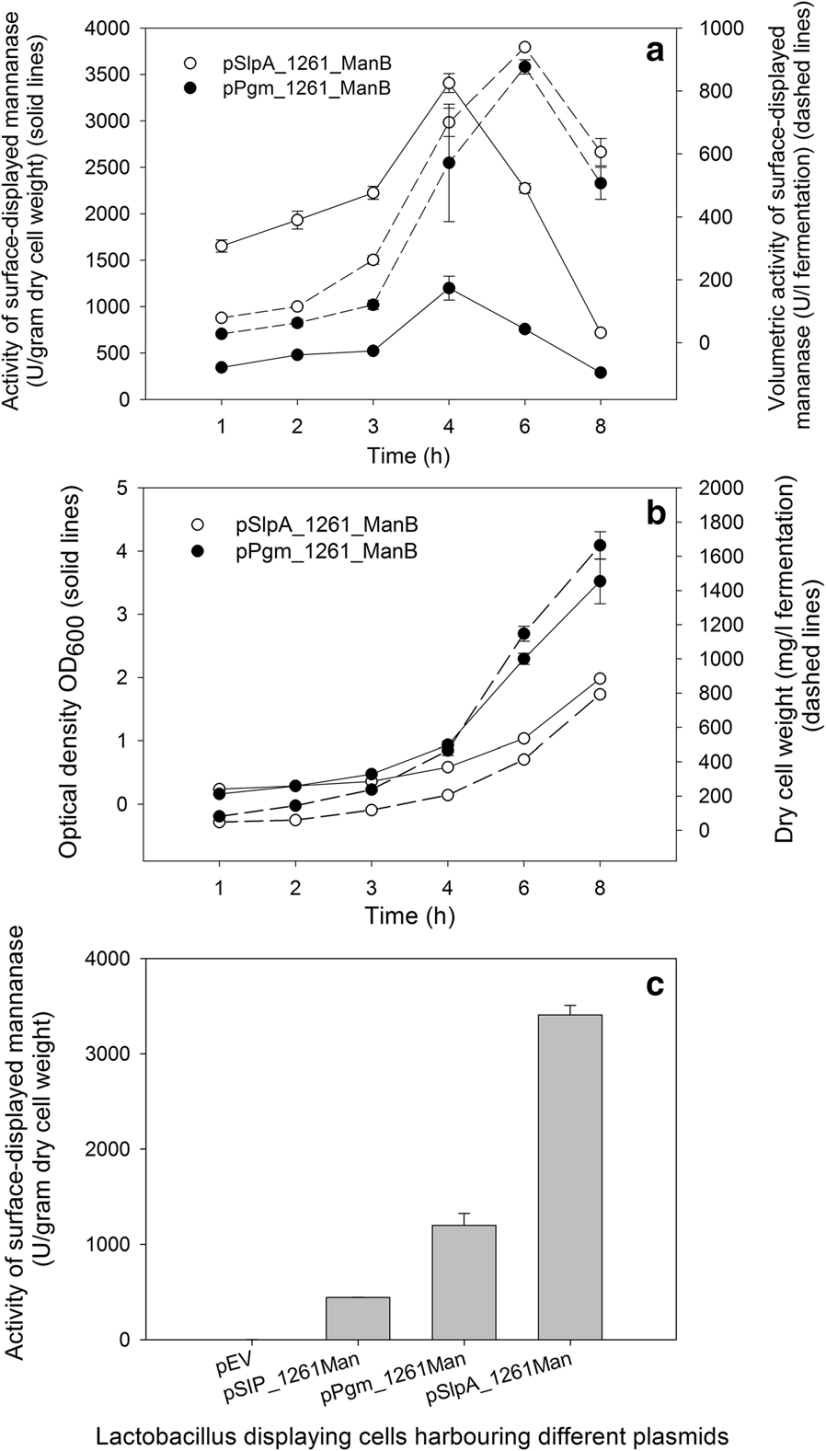
Enzymatic activity of ManB-displaying cells. **a**, **b** Time course of cultivations of ManB-displaying *L. plantarum* recombinant strains harboring the plasmids pPgm_1261ManB (closed symbols) and pSlpA_1261ManB (open symbols) in MRS medium at 37 °C with activities of surface displayed mannanase expressed as U/g dry cell weight and U/l fermentation (**a**) and OD_600_ and dry cell weights (mg/l fermentation) (**b**). **c** Maximum activities of ManB-displaying *L. plantarum* carrying various plasmids: pEV (negative control); pSIP_1261ManB for inducible expression of ManB; pSlpA_1261ManB and pPgm_1261ManB for constitutive expression of ManB

The enzyme activities of living recombinant bacteria were determined to examine the functionality of the surface-displayed enzymes. Figure 4a, b show the time course of the cultivations of ManB-displaying *L. plantarum* strains harboring the pSlpA_1261ManB or pPgm_1261ManB plasmids. The highest levels of mannanase-displaying activities (U/g dry cell weight) of both recombinant bacteria were obtained at 4 h after inoculation (Fig. 4a). A significant decrease in activities (U/g dry cell weight) of surface-displayed mannanase cells was observed as the cultivation was extended after 4 h. However, the highest volumetric activities of both recombinant strains (~ 900 U/l fermentation) were obtained at 6 h of incubation, inferring that this is the optimal time point to harvest the cells. Interestingly, *L. plantarum* harboring pSlpA_1261ManB grew slower than *L. plantarum* harboring the other constitutive promoter Pgm. At 6 h of the cultivations, the amount of dry cell weight of the strain harboring pPgm_1261ManB under control of the Pgm promoter (1150 mg/l fermentation) were approximately three-fold higher compared to the strain harboring pSlpA_1261ManB under control of SlpA promoter (410 mg/l fermentation) (Fig. 4b). The highest enzymatic activities of ManB-displaying cells were 3500 and 1200 U/g of dry cell weight, which were obtained with the strains carrying the plasmids, pSlpA_1261ManB and pPgm_1261ManB, respectively (Fig. 4c). These results confirmed that the enzymatic activities of ManB-displaying cells obtained from constitutive systems were significantly higher compared to the activities obtained from the inducible system, which was 460 U/g of dry cell weight (Fig. 4c). No mannanase activity was detected from *Lactobacillus* cells carrying the empty plasmid pEV (negative control), thus confirming that the enzymatic activities obtained from ManB-displaying cells were indeed from surface-anchored β-mannanase.

### Stability of the pSlpA_1261ManB displaying cells

**Fig. 5:**
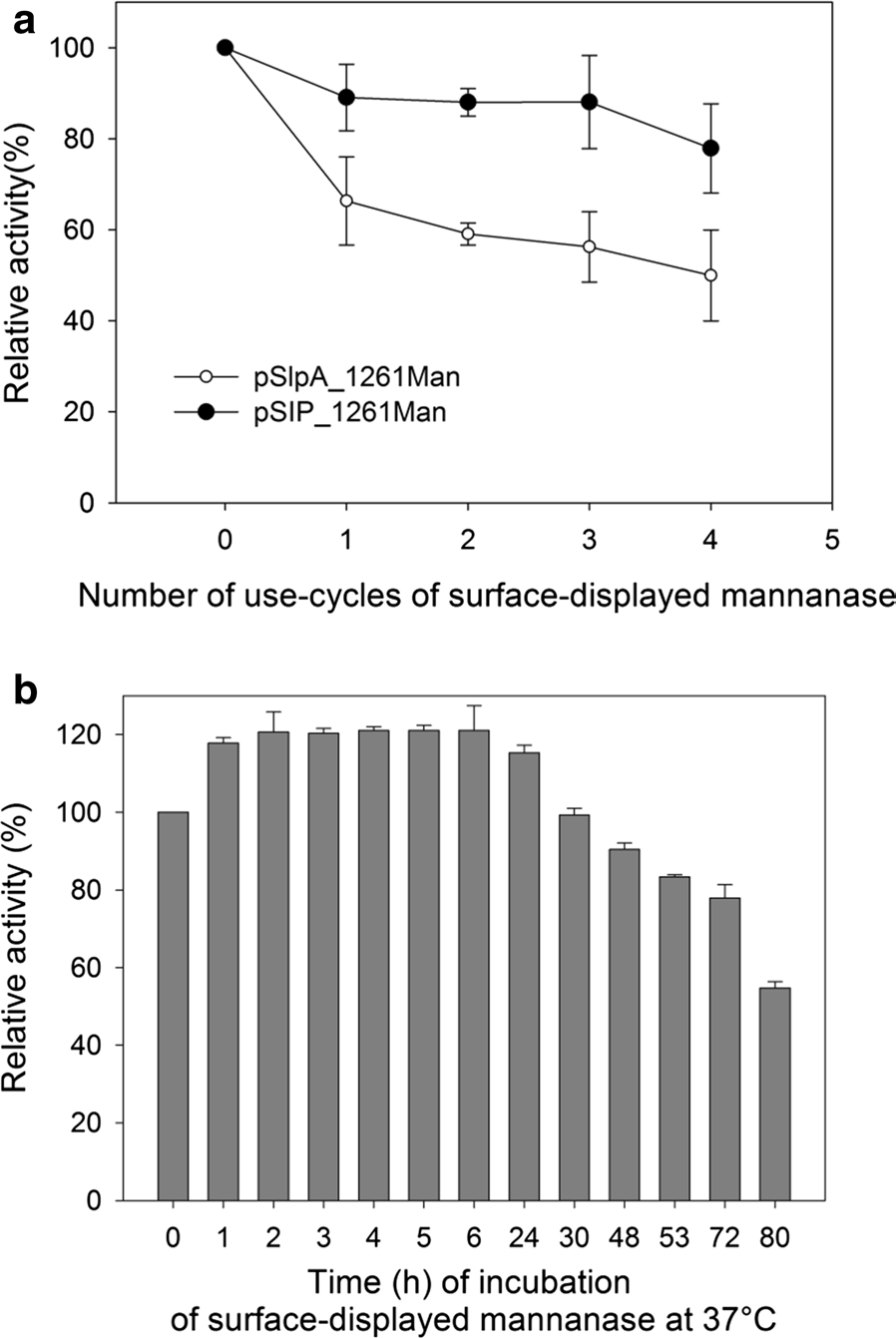
Enzyme activity of surface displayed ManB in viable *L. plantarum.* **a** Results of repeated activity measurements of *L. plantarum* cells harboring pSlpA_1261ManB in comparison with pSIP_1261ManB, with 0 indicating freshly harvested cells, while 1, 2, 3, 4 indicated the number of repetitions; **b** Enzyme activity of ManB displayed on *L. plantarum* harboring pSlpA_1261ManB at various period of incubation in PBS and at 37 °C. The activity is relative to time point 0 (freshly harvested cells)

Due to clear confirmation of surface localization and higher enzymatic activity of ManB-displaying cells, the strain harboring pSlpA_1261ManB was chosen for further characterization. In order to test the reusability of ManB-displaying cells, the enzymatic activity of cells harboring pSlpA_1261ManB was measured at 37 °C during four repeated cycles with extensive washing between cycles to remove proteins released by lysis cells. *L. plantarum* cells harboring pSlpA_1261ManB retained 51% residual activity after four assay/washing cycles, which was lower in comparison to the *L. plantarum* cells harboring pSIP_1261ManB with an inducible promoter (Fig. 5a). ManB-displaying cells carrying pSIP_1261 ManB retained 78% of its initial activity after 4 cycles (Fig. 5a). Furthermore, the activity of the ManB-anchored cells was stable during 30 h of incubation at 37 °C (Fig. 5b), in fact, the enzyme showed higher activity in the first 24 h after being collected from the cultures and re-suspended in PBS buffer, which might be due to the presence of cations in PBS buffer and their effects on mannanase activity. Presence of K^+^ has been previously reported to slightly activate mannanase activity [28, 29]. ManB-displaying cells retained 80% and 50% of initial mannanase activity after 72 h and 80 h, respectively, at 37 °C.

### Analysis of formed manno-oligosaccharides (MOS)

**Fig. 6:**
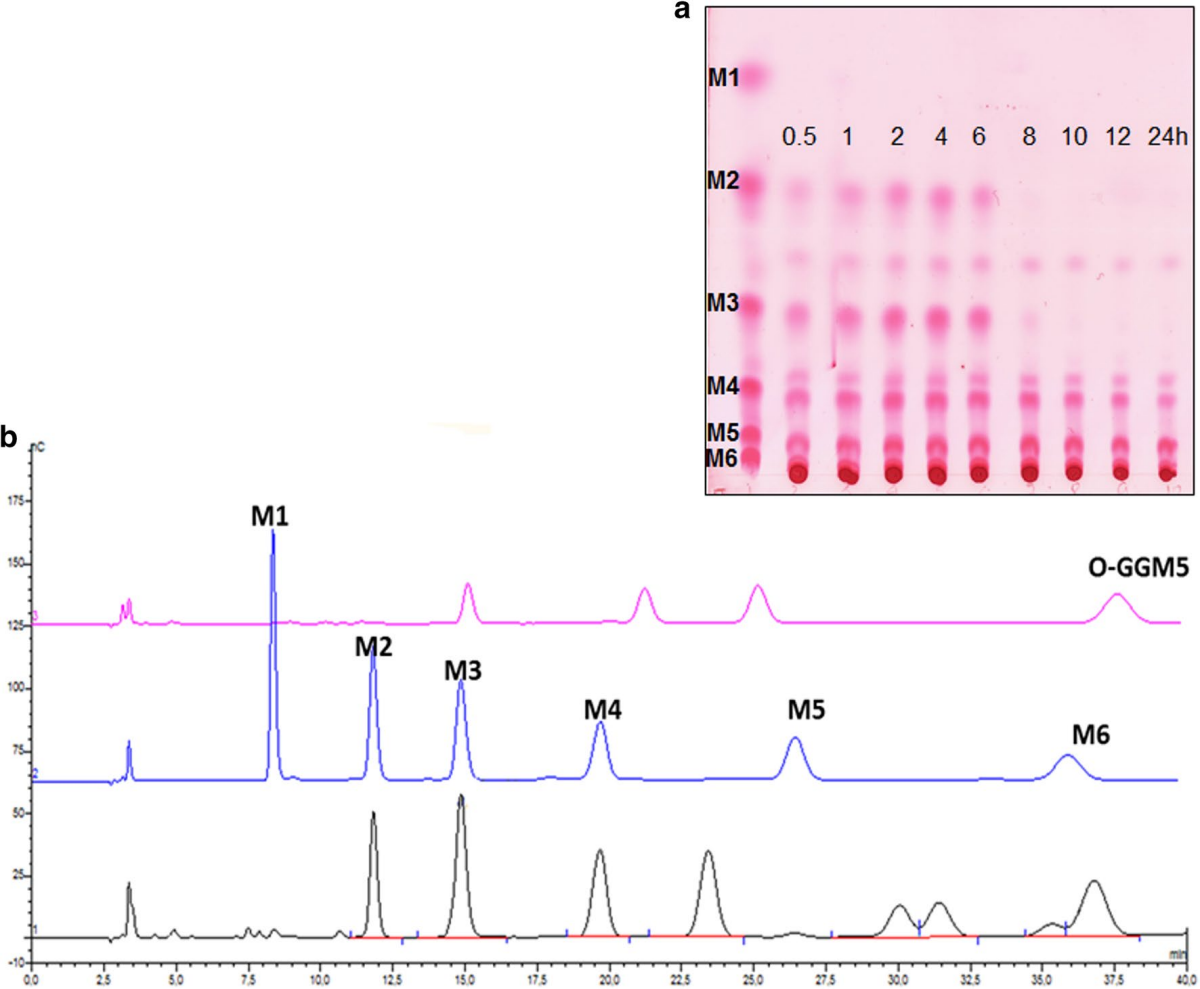
Formation of manno-oligosaccharides (MOS) from LBG (5%) by mannanase-displaying *L. plantarum* cells harboring pSlpA_1261ManB at 37 °C. **a** Thin layer chromatography (TLC) analysis; Standards: mannose, M1, mannobiose, M2, mannotriose, M3, mannnotetraose (M4), mannopentose (M5), mannohexaose, M6. **b** HPAEC chromatogram of the reaction mixture after 4 h of conversion (black line); Standards: *O*-GGM5 (pink line), M1–M6 as used in TLC analysis (blue line)

The conversion of 5% (w/v) LBG in 50 mM sodium citrate buffer (pH 6.0) using the recombinant *L. plantarum* cells harboring pSlpA_1261ManB was carried out at 37 °C. The ManB-displaying cells were added to a final enzyme activity of 50 U/ml of reaction mixture. Analysis of product formation by TLC showed that various manno-oligosaccharides (M2 to M6) were formed (Fig. 6a). Interestingly, the presence of smaller MOS such as mannobiose (M2), mannotriose (M3) was not observed after 8 h of the conversion. The analysis of product mixture by HPAEC-PAD supported the observation. The highest MOS yield of LBG conversion was 14% after 4 h, of which 1.2 mM of mannobiose, 2.2 mM of mannotriose, 3.0 mM of mannotetraose, 0.7 mM of mannohexaose and 3.6 mM of *O*-GGM5 were determined (Fig. 6b). The results revealed that surface-displayed mannanase is able to degrade galactomannan (LBG) into MOS, and smaller MOS were likely metabolized by *Lactobacillus* cells during cultivation.

## Discussion

*Lactobacillus plantarum*, a well-studied Gram-positive bacterium belonging to the group of lactic acid bacteria (LAB), is of great interest in food fermentation applications because of its GRAS (generally recognized as safe) status and probiotic characteristics [30, 31]. This bacterium can be found as beneficial inhabitant of the human gastrointestinal (GI) tract, where it is able to persist for up to 7 days [12]. In probiotic bacteria certain adherence factors are believed to play an important role in their stimulating interactions with the host, including their persistence in the GI tract and the exclusion of pathogens by competition [30, 32]. In addition, *L. plantarum* possesses a rigid structure of a thick cell wall and high acid tolerance [33]. Because of these benefits, *L. plantarum* has been exploited not only as food-grade cell factory [34–36], but also as a vehicle for in situ delivery of bioactive compounds to the human GI-tract [27, 37, 38].

The development of *L. plantarum* as whole cell biocatalyst is of our interest using the approach of displaying active enzymes on the cell surface, in which enzyme molecules are produced and anchored on the bacterial cell surface simultaneously, and the living whole cells can be used directly after cultivation and harvest [19, 22]. Moreover, the bacterial cells displaying target enzymes can be stored and re-used when needed. This would greatly reduce the cost of biocatalyst preparation and downstream processing compared to traditional methods. Hence, the improvement of genetic tools for such ‘genetic’ immobilization of enzymes in lactobacilli would facilitate further development of *L. plantarum* in several applications.

We have recently developed *L. plantarum* as a carrier for mannanase displayed at the cell surface using a lipoprotein anchor (Lp_1261) and exploited the cells for production of health-promoting and potentially prebiotic oligosaccharides MOS [22]. Notably, the anchoring system was developed based on the strong inducible lactobacillal pSIP expression system [39, 40] which is specific for *Lactobacillus* hosts, and has been proven to offer high expression levels and tight control by externally added nano-gram amounts of the inducer (IP-673) [41]. Inducible systems are preferred when expressing, e.g. proteins that have toxic or otherwise adverse effects on the host. When develop a biocatalyst using bacterial cells it is more convenient to use constitutive expression. It is also an economical perspective and carefully monitoring the inducing point for the gene expression is not necessary.

In this study, we demonstrated the use of two constitutive promoters (Pgm, SlpA) in the expression vectors for surface display of mannanase using the lipoprotein anchor Lp_1261 in *L. plantarum*. Western blotting confirmed the successful expression of *manB* gene under control of both promoters in *L. plantarum*. However, using the SlpA promoter clearly resulted in higher production of mannanase compared to the Pgm promoter (Fig. 2). It is interesting to mention that the promoter region of the *L. acidophilus slpA* gene expression site consists of two sub-promoters P-1 and P-2 [26]. However, only sub-promoter P-1 was employed to direct mRNA synthesis. Computer analysis indicated that the untranslated leader sequence of the mRNA, which is directed by sub-promoter P-1, can fold into a large stem-loop structure that may protect mRNA from degradation [26]. This might be the reason why the protein production of pSlpA_1261Man harboring cells was higher than that of pPgm_1261Man. The flow cytometry and fluorescence microscopy analysis (Fig. 3) also reflected higher number of surface displayed mannanase using SlpA compared to the Pgm promoter. The growth rate of the strain harboring plasmid with the SlpA promoter was lower than with the Pgm promoter. This indicates that high protein production using the SlpA promoter induce metabolic burden in the host, which is a common consequence of high production of secreted proteins in Gram-positive bacteria [42, 43]. In addition, the surface of *Lactobacillus* cells is covered with different proteins and exopolysaccharides (EPS) with the growth of cells, thus cell density is important for surface display system as these components may have an impact on the surface displayed heterologous proteins and their activity. This might explain that the slower growth of the strain harboring plasmid with the SlpA promoter results in a higher activity of surface displayed mannanase.

The surface localization of ManB was not clearly confirmed by flow cytometry and immunofluorescent microscopy for the strain carrying the plasmid pPgm_Lp1261ManB. We speculate this could be the attachment of the N-terminus of the lipoprotein anchor to the plasma membrane. The Myc-tag of ManB in the case of Pgm_1261ManB might be buried in the cell wall and thus not sufficiently exposed for recognition by the antibody used for Myc detection. However, the activity of surface-displayed ManB was detected for the strain carrying the plasmid pPgm_Lp1261ManB and the highest activity was found to be 1200 U/g dry cell weight (Fig. 4a). This is approximately 3-fold lower than the level obtained with the strain carrying pSlpA_1261ManB, but more interestingly, 2.6-fold higher than the level obtained from the strain carrying the inducible plasmid pSIP_1261ManB (460 U/g dry cell weight). Not surprisingly, the *Lactobacillus* strain harboring pSlpA_1261ManB showed significantly lower dry cell weight yield and slightly higher volumetric activity of surface-displayed mannanase than the strain harboring pPgm_1261ManB. This implies that the amount of active mannanase (i.e., the number of enzyme molecules) displayed on the cell surface of the strain under control of the SlpA promoter was higher than the strain under control of the Pgm promoter. In addition, based on the known specific activity of the purified soluble ManB (1800 U/mg) [5], the amounts of active surface-anchored ManB expressed under the direction of Pgm and SlpA are roughly estimated to be 0.6 and 1.9 mg/g dry cell weight, respectively, which are significantly higher than the amount obtained from inducible system (0.25 mg/g dry cell weight).

## Conclusion

We have demonstrated constitutive expression and display of a β-mannanase on the cell surface of *L. plantarum* with the use of strong constitutive promoters, which will continuously drive synthesis of the recombinant protein without the need to add an inducer or change the growth conditions of the host strain. Our approach, where the enzyme of interest is displayed on the cell surface of a food-grade organism, should result in the availability of safe, stable food-grade biocatalysts that can be used in different production processes relevant for food industry in a more efficient and sustainable way. This system could also be of interest for in situ production and delivery of proteins to human and animal host.

## Materials and methods

### Chemicals, enzymes and plasmids

All chemicals were purchased from Sigma-Aldrich (St. Louis, MO) unless stated otherwise and were of highest purity available. Restriction enzymes, polymerases, T4 DNA ligase and corresponding buffers were obtained from Fermentas (Vilnius, Lithunia) and used as recommended by the manufacturer. The plasmids pSIP_1261ManB [22] containing the mannanase gene (*manB*) from *B. licheniformis* DSM13 (ATCC 14580) and pUC57 (provided by GenScript) containing the synthetic constitutive promoter *pgm* from *L. acidophilus* NCFM were used as the templates for the amplification of ManB and the Pgm promoter. All plasmids used in this study are listed in Table 1.

**Table 1:** Strains and plasmids used in this study

Strain or plasmid	Relevant characteristic (s)	References
Strains		
*L. acidophilus* ATCC4356	Source of constitutive promoter P_*slpA*_	DSMZ (Brauschweig, Germany)
*L. plantarum* WCFS1	Host strain	[30]
*E. coli* NEB5α	Host strain	New England Biolabs
Plasmids		
pSIP409GusA	Erm^r^; *spp*- based expression vector pSIP409	[40]
pEV	Erm^r^; pLp_2578sAmyA derivative, no signal sequence, no *man* (negative control)	[27]
pUC57	Amp^r^; plasmid cloning vector in *E. coli* containing the synthetic constitutive promoter *pgm*	GenScript
pSIP_1261ManB	Erm^r^; pLp_1261InvS derivative with fragment of *man*-*myc* instead of the *inv* gene	[22]
pPgm_1261ManB	Erm^r^; pSIP_1261ManB derivative with fragment of constitutive promoter P_*pgm*_ instead of inducible promoter P_*sppQ*_ in pSIP409 vector	This study
pSlpA_1261ManB	Erm^r^; pSIP_1261ManB derivative with fragment of constitutive promoter P_*slpA*_ instead of inducible P_*sppQ*_ in pSIP409 vector	This study

### Bacteria strains, media and culture conditions

The bacterial strains used in this study are listed in Table 1. *L. acidophilus* ATCC4356 was purchased from DSMZ (Brauschweig, Germany). *L. plantarum* WCFS1, isolated from human saliva as described by Kleerebezem et al. [30], was originally obtained from NIZO Food Research (Ede, The Netherlands) and maintained in the culture collection of the Norwegian University of Life Sciences, Ås, Norway. *Escherichia coli* NEB5α (New England Biolabs (NEB), Frankfurt am Main, Germany) was used in the transformation experiments involving the subcloning of DNA fragments and was grown in Luria-Bertani (LB) broth at 37 °C with shaking at 120 rpm. *L. plantarum* was grown in deMan, Rogosa and Sharpe (MRS) broth (Oxoid) at 37 °C without agitation. When needed, erythromycin was supplemented to media in concentrations of 200 µg/ml and 5 µg/ml erythromycin for *E. coli* and *L. plantarum*, respectively. Solid media were prepared by adding 1.5% (w/v) agar to the broth.

### DNA manipulation

Plasmids were isolated from *E. coli* strains using the PureYield™ plasmid miniprep System (Promega). PCR amplifications of DNA were done using proof-reading Phusion polymerase (NEB). The primers used in this study, which were purchased from VBC Biotech (Vienna, Austria), are listed in Table 2. The sequences of PCR-generated inserts were verified by DNA sequencing performed by a commercial provider (Microsynth; Vienna, Austria). PCR products and DNA fragments obtained by digestion with restriction enzymes were purified using the Illustra™ GFX™ PCR DNA and Gel Band Purification Kit (GE Healthcare, UK). Ligations were performed using T4 DNA ligase (Fermentas; Vilnius, Lithuanis). All plasmids were transformed into *E. coli* NEB5α chemical competent cells following the manufacturer’s protocol for obtaining the plasmids in sufficient amounts. The constructed plasmids were transformed into electrocompetent cells of *L. plantarum* WCFS1 according to the protocol of Aukrust and Blom [44].

**Table 2:** Primers used in this study

Primer	Sequence^a,b^ (5′ → 3′)	Restriction sites
M1 (Fw-pgm)	ATGCAGATCT*TGCGACAAGTAATAAACTAAAC*	*Bgl*II
M2 (Rv-pgm1261Man)	*CTTTTGCAGCTGTTTTGAAATTCATAGCCTTCTTAGCTTCTTCAAC*	
M3 (Fw-pgm1261Man)	*GTTGAAGAAGCTAAGAAGGCTATGAATTTCAAAACAGCTGCAAAAG*	
M4 (Rv-1261Man)	ATGCGAATTC*TTACAGATCCTCTTCTGAGATG*	*EcoR*I
M5 (Fw-slpA)	ATGCAGATCT*ATAAAGTTGTTTGATAAATGCTCAAC*	*Bgl*II
M6 (Rv-slpA1261Man)	*TGCAGCTGTTTTGAAATTCATGTGGTCTTTTCCTCC*	
M7 (Fw-slpA1261Man)	*GGAGGAAAAGACCACATGAATTTCAAAACAGCTGCA*	

^a^Nucleotides in italics anneal to the DNA of the target gene

^b^Introduced restriction sites underlined

### Plasmid construction

The schematic overview for the construction of the constitutive expression cassette for secretion and anchoring of the ManB is presented in Fig. 1. The anchoring sequence used in this study was taken from pSIP_1261ManB (Table 1), which is a derivative of the pLp_1261InvS vector that has been developed for inducible gene expression and anchoring in lactobacilli [27]. The plasmid contains a N-terminal signal peptide (SP) derived from the gene encoding a lipoprotein anchor, Lp_1261, which contains 75 residues in total including 22 amino acids of the SP. The C-terminus of the target gene, *manB*, was fused to a 30-bp fragment encoding the *myc* tag (GAACAAAAACTCATCTCAGAAGAGGATCTG), as shown in Fig. 1.

For construction of the plasmid pPgm_1261ManB the two fragments, *pgm* (~ 357 bp) and *lp1261*-*manB*-*myc* (~ 1299 bp), were PCR-amplified using the plasmid pUC57 and the plasmid pSIP_1261ManB, respectively, as the templates with two primer pairs, which were M1/M2 and M3/M4 (Table 2), respectively. The two resulting fragments were fused by overlap extension PCR, which consists of two PCR steps. The first PCR step was performed without added primers under the following conditions: initial denaturation at 98 °C for 30 s followed by 15 cycles of denaturation at 98 °C for 10 s, annealing at 65 °C for 20 s, extension at 72 °C for 1 min, and additional 5 min elongation at 72 °C. In the second PCR step, the primer pair M1/M4 was used to amplify the whole insert containing an N-terminal *Bgl*II site and a C-terminal *EcoR*I site. The primers were added to the reaction tubes followed by 20 cycles as described in the first PCR step but the annealing temperature was at 51 °C. Subsequently, the fused fragment containing *pgm*-*lp1261*-*manB*-*myc* (~ 1657 bp) was ligated into the *Bgl*II-*EcoR*I digested vector pSIP409GusA (~ 5.4 kb), yielding the plasmid pPgm_1261ManB.

For construction of the plasmid pSlpA_1261ManB, a similar cloning strategy was applied. The primer pairs M5/M6 and M7/M4 (Table 2) were used to amplify two fragments, *slpA* (~ 554 bp) and *lp1261*-*manB*-*myc*, using genomic DNA from *L. acidophilus* ATCC4356 and the plasmid pSIP_1261ManB as templates, respectively. The two resulting fragments were fused by overlap extension PCR as described above and the primer pair M5/M4 was used to amplify the whole insert containing an N-terminal *Bgl*II site and a C-terminal *EcoR*I site. The fused fragment containing *slpA*-*lp1261*-*manB*-*myc* (~ 1854 bp) was ligated into the *Bgl*II-*EcoR*I digested vector pSIP409GusA (~ 5.4 kb), yielding the plasmid pSlpA_1261ManB. The resulting constitutive expression vectors were maintained in *E. coli* NEB5α before being transformed into the expression host *L. plantarum* WCFS1.

### Constitutive gene expression in *L. plantarum*

The plasmids pPgm_1261ManB and pSlpA_1261ManB were transformed into electro-competent *L. plantarum* WCFS1 and transformants were selected on MRS agar plates containing 5 μg/ml erythromycin. For gene expression, overnight cultures of *L. plantarum* WCFS1 harboring the novel plasmids with constitutive promoters were diluted in 50 ml of fresh pre-warmed MRS broth containing 5 µg/ml of erythromycin to a cell density with an OD_600_ of 0.1 and incubated at 37 °C without agitation. The cells were harvested after 4 h at an OD_600_ of approximately 0.6–0.9 by centrifugation (4000×*g*, 10 min at 4 °C) and washed with phosphate buffered saline PBS (137 mM NaCl, 2.7 mM KCl, 2 mM KH_2_PO_4_, 10 mM Na_2_HPO_4_, pH 7.4) and then re-suspended in 1 ml of PBS containing 20 µl of 50 mM PMSF. For a positive control (the cells harboring pSIP_1261ManB), gene expression was induced at an OD_600_ ~ 0.3 by adding the inducing peptide pheromone IP-673 [39, 45] to a final concentration of 25 ng/ml, and the cells were harvested 2 h after induction at an OD_600_ of approximately 0.8–1.2 by centrifugation (4000×*g*, 10 min at 4 °C) [39]. The disruption of the cells was performed with ~ 1 g of glass bead (0.1 mm) using the Precelly 24 glass bead mill (PEQLAB Biotechnology GmbH, Erlangen, Germany). Cell-free extracts (crude extracts) obtained after 5 min of centrifugation at 10,000×*g* and 4 °C were used for Western blot analysis.

### Gel electrophoresis and western blotting

Cell-free extracts (crude extracts) obtained from 50 ml culture were freshly prepared prior to electrophoresis on SDS-PAGE gels (10% acrylamide). Proteins (~ 5.5–6.0 mg/ml of total protein of each sample) on the SDS-PAGE gels were transferred to a nitrocellulose membrane using the Trans-Blot Turbo Transfer system (Bio-rad Laboratories, Inc) according to the manufacturer’s recommendation. Nonspecific protein interactions were blocked by incubating the membrane with 50 ml of 1% BSA dissolved in Tris-buffered saline-Tween 20 (TBS-T) for 1 h on the shaker at room temperature. The membrane was immediately incubated with monoclonal murine anti-Myc antibody (Invitrogen) in TBS-T containing 0.5% BSA overnight at 4 °C with shaking. The membrane was then rinsed three times with 15 ml of TBS-T before being incubated with a secondary antibody, which was a polyclonal rabbit anti-mouse antibody conjugated with horseradish peroxidase (HRP) (Dako, Denmark), and Precision Protein™ Streptactin-HRP Conjugate (Bio-Rad) for 1 h at room temperature. Before visualization, the membrane was again rinsed three times with 15 ml of TBS-T, following by incubating with SuperSignal West Pico chemiluminescent substrate (Bio-Rad). The protein bands were visualized by the Chemidoc™ XRS + imaging system (Bio-Rad).

### Flow cytometry and indirect immunofluorescence microscopy analysis

The staining of cells for flow cytometry and indirect immunofluorescence microscopy analysis was carried out as previously described [22, 38] with some modifications. One ml of cell cultures (OD_600_ of ~ 0.5) was harvested after 4 h of incubation and cells were re-suspended in 50 µl PBS containing 1% of BSA (PBS-B) and 0.4 µl of monoclonal anti-Myc antibodies (Invitrogen; diluted 1:5000 in PBS-B). After incubation at RT for 30 min, the bacterial suspension was centrifuged at 5000×*g* for 3 min at 4 °C and washed three times with 500 µl PBS-B. The cells were subsequently incubated with 50 µl of PBS-B and 0.8 µl of Goat anti-mouse IgG H&L/Alexa Flour 488 (Abcam, Cambridge, UK, diluted 1:2500 in PBS-B) for 30 min at RT, in the dark. After collecting the cells by centrifugation (4000×*g*, 3 min at 4 °C) and washing five times with 500 µl PBS, the stained cells were analyzed by flow cytometry using a MACSQuant analyser (Miltenyi Biote, Bergisch Gladbach, Germany), following the manufacturer’s instructions. For indirect immunofluorescence microscopy, the stained bacteria were visualized under a Leica TCS SP5 II confocal laser scanning microscope (Leica Microsystems, GmbH, Mannheim, Germany) using the 488-nm argon laser line. The fluorescence detection window was set between 505 and 550 nm. Images were acquired with a PL APO 63×/1.40 oil immersion objective.

### Enzyme assay

Enzymatic activities were determined as described previously [10, 22, 46]. The reaction mixtures consisted of 100 µl of a suspension the mannanase-displaying cells in PBS and 900 µl of a 0.5% (w/v) galactomannan solution (locust bean gum, LBG; Megazyme, Bray, Ireland). The galactomannan solution was prepared by dissolving LBG in 50 mM sodium citrate buffer (pH 6.0) at 50 °C for 30 min.

Enzyme-displaying cells were collected from the cultures 4 h after inoculation by centrifugation at 4000×*g* for 5 min at 4 °C. Cell pellets obtained from 50 ml culture were washed twice with PBS and re-suspended in 100 µl of PBS. The mannanase-displaying cells were incubated with LBG solutions, respectively, at 37 °C with mixing at 600 rpm for 5 min. The cells were removed by centrifugation (5000×*g*, 4 °C, and 2 min) and the amount of reducing sugars released in the supernatant of the enzymatic reaction was determined by the dinitrosalicylic acid (DNS) assay. Briefly, 100 µl of the reaction supernatant were mixed with 100 µl of DNS solution, followed by heating at 99 °C for 10 min, cooling on ice for 5 min, and diluted with 800 µl of de-ionized water, before measuring the absorbance at 540 nm using 1–5 µmol/ml of d-mannose as standard. One unit of mannanase activity was defined as the amount of enzyme releasing 1 µmol of mannose equivalents per minute under the given conditions. The reactions were done in triplicates and the standard deviations were always < 5%.

### Stability of β-mannanase displaying cells

Cells obtained after the incubation with substrate were washed with 500 µl of PBS and collected by centrifugation (5000×*g*, 4 °C, 2 min). The cells were then re-suspended in 100 µl of PBS and mannanase activities were measured as described above. This procedure was repeated for several cycles of activity measurements with intermediate washing steps to evaluate the number of use cycles of surface displayed β-mannanase.

In order to determine the catalytic stability of β-mannanase displaying cells at 37 °C, the cells were collected from the cultures 4 h after inoculation and re-suspended in 100 µl of PBS prior to incubation at 37 °C. At certain time intervals, enzymatic activity of ManB-displaying cells was measured using LBG 0.5% as the substrate under standard assay conditions. Residual activities (*A*_*t*_/*A*_*0*_, where *A*_*t*_ is the activity measured at time t and *A*_*0*_ is the initial activity) were plotted versus the incubation time. The half-life time of activity was determined when residual activity reaches 50%.

### Locust bean gum (LBG) conversion

Conversion of 5% (w/v) LBG, which were prepared in 50 mM sodium citrate buffer at pH 6.0, catalysed by surface displayed mannanase was carried out on a 2-ml scale with 100 U of mannanase activity, which was obtained from the expression strain harboring the plasmid pSlpA_1261ManB, at 37 °C for 24 h. Agitation was applied at 150 rpm and the samples were taken at regular intervals. The reactions were stopped by heating the samples at 100 °C for 5 min.

### Thin-layer chromatography (TLC)

TLC was performed by high performance TLC silicagel plates (Kiselgel 60 F245, Merck). Appropriately diluted samples were applied to the plate (0.5 µl) and eluted twice in ascending mode with a iso-propanol/n-butanol/water mixture (12:4:5). Thymol reagent was used for visualization. A mixture of M1-M6, which contains mannose, mannobiose, mannotriose, mannotetraose, mannopentose and mannohexaose, (Megazyme, Ireland) was used as standards.

### High performance anion exchange chromatography (HPAEC)

Separation of the oligosaccharides released from locust bean gum during the conversion described above was carried out using high performance anion exchange chromatography with pulsed amperometric detection (HPAEC-PAD). Separation was performed at 30 °C on a CarboPac PA-1 column (4 mm × 250 mm) connected to a CarboPac PA-1 guard column (Dionex). For separation of manno-oligosaccharides, eluents A (150 mM NaOH) and B (100 mM NaOH and 0.5 M NaOAc) were mixed to form the following gradient: 100% A for 40 min, 100% B for 5 min, and then 100% A for another 15 min. The column was washed with 100% B for 10 min and re-equilibrated for 15 min with the starting conditions of the employed gradient. Manno-oligosaccharides standards are mannobiose, mannotriose, mannotetraose, mannopentose, mannohexaose and 63, 64-α-d-galactosyl-mannopentaose (*O*-GGM5) (Megazyme).
